# Effect of social robots in reducing loneliness among community-dwelling older adults: A mixed-method approach

**DOI:** 10.1093/geroni/igaf122.3049

**Published:** 2025-12-31

**Authors:** Hiroshi Murayama, Mai Takase

**Affiliations:** Tokyo Metropolitan Institute for Geriatrics and Gerontology, Tokyo, Japan; Tokyo Metropolitan Institute for Geriatrics and Gerontology, Itabashi City, Tokyo, Japan

## Abstract

Most studies on interventions using social robots to reduce loneliness have been conducted in facilities in Western nations. This study evaluated the effectiveness of social robot interventions in reducing loneliness among community-dwelling older Japanese adults using a randomized controlled trial and qualitative analysis. Individuals aged ≥65 years who lived alone in Tokyo and neighboring areas and experienced loneliness were recruited. Seventy-three eligible participants were randomly assigned to either an intervention or control group. The four-week intervention involved a humanoid social communication robot (BOCCO emo), which facilitated conversations with human operators and/or family members and reminded participants of daily tasks. The primary outcome was loneliness, with psychological well-being, depression, and laughter frequency as secondary outcomes. Participants were evaluated at baseline and follow-up. In the follow-up survey, participants in the intervention group provided open-ended responses regarding their experiences using the social robot. A total of 68 participants completed both the baseline and follow-up surveys (n = 34 in each group; mean age: 82.3 years; 94.1% women). Loneliness decreased more in the intervention group than in the control group. Psychological well-being improved, and the frequency of laughter tended to increase in the intervention group. Content analysis identified four categories: “emotional support and psychological connection,” “lifestyle assistance,” “enrichment of social interaction,” and “cognitive and mental stimulation.” Social robots can reduce loneliness among community-dwelling older adults in non-Western societies. Information and communication technology appears to be an effective approach to alleviating loneliness and enhancing well-being among older adults in community settings.

